# Dangers Which Threaten the Use of Cod Liver Oil

**Published:** 1896-06

**Authors:** Jno. T. Winter

**Affiliations:** Professor of Theory and Practice of Medicine, National University, Washington, D. C.


### Southern Medical Record.

A MONTHLY JOURNAL OF MEDICINE AND SURGERY.

VOL. XXVI. ATLANTA, GA., JUNE, 1896. No. 6.

### Original Articles.

### DANGERS WHICH THREATEN THE USEFULNESS OF COD-LIVER OIL.

BY JNO. T. WINTER, M. D.,

Professor of Theory and Practice of Medicine, National University, Washington, D. C.

From recent observations I am convinced there are dangers which threaten the future usefulness of one of the most valuable remedies in the materia medica.

That cod-liver oil has valuable therapeutic properties has been known for two centuries. For half a century this oil has been carefully studied, and its physiological action and therapeutics placed upon a sound, scientific basis. Until recently, thousands of physicians have prescribed cod-liver oil, confident that certain results would follow. Thousands upon thousands of the laity have taken advantage of the teachings of the profession and the experience of their fellow-men, and incalculable benefit has resulted therefrom.

It is no slight matter to weaken the confidence of the profession in a remedy which has stood the test for at least fifty years. It is equally serious to diminish the faith which the people have in so valuable a remedy. No one can deny the fact that cod-liver oil has given comfort to those who could not be cured, has greatly prolonged the lives of vast numbers and has restored countless thousands to health.

In the light of all this, is it not trifling with human life to break the faith of both physician and patient in a remedy which has proven itself so valuable, we might say indispensable? Is the profession soon to lose faith in cod-liver oil as a remedial agent? And is the public about to abandon its use?

Neither physician nor patient will continue the use of a remedy unless the desired results are obtained. Experience has established these results. Cod-liver oil is not prescribed as an experiment. Its physiological action is no longer watched with an uncertain eye. But suppose these expected results no longer appear, what then? Confidence is impaired, and in looking about for a substitute a hundred worthless remedies are tried, and the most valuable time for successful treatment is lost.

Having for many years been a firm believer in the curative properties of cod-liver oil, I have of late looked with great anxiety on efforts which tend to impair its usefulness. I can see at least three great dangers. Unless these are destroyed, the therapeutic value of cod-liver oil will be an uncertain factor.

THE FIRST DANGER.—The first danger is the result of a modern theory that a part equals the whole! We all know that cod-liver oil is a most complex body. Its chemical analysis reveals the presence of many constituents. These have been called the alkaloids, or the "active principle" of the oil. They exist in peculiar combination with the fat, and are with difficulty separated from it. It is significant that nearly every original investigator has been able to extract from the oil some additional substance. In fact, even prior to 1888 there had been discovered over fifty elements in this oil. Within the past eight years chemists have been very active in discovering new principles, until at the present time I am not aware of the length of the list.

To break up cod-liver oil into these several ingredients is to change its character altogether. I do not believe it is possible to take any of these constituents from the oil without either impairing or totally destroying the therapeutic properties of the parts taken or of those remaining. There is no doubt but the alkaloids of cod-liver oil are valuable; they are valuable, however, *because of the peculiar combination as it exists in the oil.* To take all of the oil or fat from cod-liver oil and then claim that the alkaloids are useful as a fat-producing food, is just as reasonable as it would be to administer iodine, bromine, or phosphoric acid to a patient as a food! Persons can live on cream and on other fatty foods, provided their digestive powers can properly care for so rich a diet. But they could not live on iodine or any of the alkaloids or active principles of cod-liver oil, or on all of them combined. To use only the alkaloids of cod-liver oil, is to employ a remedy without value as a fat-producing food. If this be not true, then let us abandon the oil entirely and rely upon other sources for our iodine, bromine, etc.

This new theory of substituting a part for the whole is, to my mind, the greatest danger which threatens the usefulness of cod-liver oil. No matter how ingeniously the alkaloids may be combined with wine or other liquids, the results of the past will not be verified by the substitution, and the profession and laity alike will distrust it. If they were only skeptical regarding these new preparations, the case might be different. But having been taught that cod-liver oil is valuable only for its alkaloids, then the failures are charged against the oil as a whole, instead of against a part.

Even granting, for argument's sake that all of the good properties of cod-liver oil reside in these so-called alkaloids the fact remains there is no reason for believing that they would exert the same good office when thus isolated as when they existed in their natural state; just as when a mineral water would not do the same amount of good if its various constituents were extracted and administered separately.

It must be laid down as a rule, founded upon scientific study and verified by scores of years of observation, upon hundreds of thousands of cases, that the *whole oil must be used* if the best therapeutic results are desired.

THE SECOND DANGER.—The second danger would be more serious than the first, but fortunately it can never grow to such magnitude. There are only a few manufacturers so unscrupulous as to impose such frauds upon the public as these to which I now refer.

In his "Practical Therapeutics," Hare says: "One of the emulsions widely advertised in the street cars of Philadelphia as 'tasteless' has been shown to contain no oil at all." He adds also that, "Oil devoid of smell is probably devoid of medicinal value, as all the peculiar properties have been purified out of it." Patients invariably object to the odor and taste of raw cod-liver oil, so it is but natural they should be pleased with these "odorless and tasteless" preparations. That these preparations are in use at all is probably due to two reasons: (1) Only a few physicians are aware of the fact that they contain no cod-liver oil whatever; and (2) the laity have no reason for doubting that the mixture is other than what it pretends. The physician and patient have every right to expect that a "preparation" of cod-liver oil contains that oil. It is the most cruel kind of deception to thus impose upon those who place all their hope for health on this valuable remedy. As it is not present, so the results are not obtained. Therefore, cod-liver oil is put down as of no value whatever in such cases. Even if the deception be discovered, it is generally too late for the oil to accomplish good, at least to its full amount.

THE THIRD DANGER.—The third danger is a universal one, and is of great importance. To understand it a few words of explanation are necessary.

Without doubt, the great bulk of cod-liver oil is taken in the form of an emulsion. The reasons for this are evident. A good emulsion has little of the fishy odor and taste, although these are always present. An emulsion is more easily borne by the stomach and is not liable to cause eructations of gas. This is probably due to the fact that when an oil is mulsified it is, in a measure, digested, for the digestion of an oil is simply breaking it into minute globules. Modern physiology teaches that the oils are not saponified, but emulsified, by the pancreatic juice; this is the case, at least, with those oils which are assimilated. The oil in ordinary emulsions probably requires some further digestion in order to prepare it for absorption; but its emulsification is doubtless a great aid to its digestion. Then again, emulsions are more pleasant to the taste than are the raw oils. Therefore we find the majority of physicians prescribing them, while the laity, undirected by physicians, almost invariably ask for them.

It is an unfortunate fact that there is no fixed standard for cod-liver oil emulsions. We find one pharmacist offering an emulsion of his own manufacture at a certain price, while his competitor just across the way is offering one at a third less. But the former gives thirty per cent. of oil, while the latter may give but ten, yet both preparations are "Emulsions." No physician should prescribe an "Emulsion" of cod-liver oil unless he knows the preparation contains cod-liver oil, and in definite proportions. To prescribe any "Emulsion" or "Preparation" of cod-liver oil simply because it is an emulsion, or preparation, is to express as much confidence in the curative properties of the label as in the drug.

The third danger, therefore, is one due to unreliability and uncertainty. It is easily overcome by the exercise of ordinary care that only those preparations be ordered which are reliable and of definite composition.

It is because I believe cod-liver oil to be such a valuable remedy that I call attention to some of the dangers which threaten its future usefulness. But they are dangers which we are abundantly able to remove, and which must, indeed, depart without effort if we keep clearly in mind the facts upon which cod-liver oil rests its reputation.

### A FREAK OF NATURE.

An extraordinary result of an operation said to be unique in medical history is reported from the Czech University in Prague. Prof. Maydl recently opened the abdominal cavity of a young man, aged nineteen, a scholar at the Technical College in Biunn, who had suffered since his childhood from a growth extending from the backbone downwards. The professor found between the spine and the intestines the undeveloped form of a child without a head, but with discernable extremities covered with fat, and grown over with hair. According to the suggestion of the professor the growth is a twin child, which for some reason or other grew into the lower part of the body of the child that was actually born. The theory, of course, required confirmation. The youth died on February 26, in consequence of the operation. The fœtus is ascertained to be of the female sex. It was in a sitting position, and connected by two membranes with the blood system of the youth, and in that way participated in the nourishment he received. Even hair about forty centimetres long is clearly visible at the part where the head of the twin child ought to have been, and the trunk was fifteen centimetres long when extracted. Prof. Maydl on February 2d showed the extraordinary phenomenon to an assembly of medical men in Prague, and now the young man is dead a *post mortem* examination will fully clear up this strange matter.—*Indian Lancet.*

